# Characterization of mpox transmission in the household, the role of children, the impact on pregnancy, and the association with lactation: a study protocol

**DOI:** 10.11604/pamj.supp.2025.50.1.47419

**Published:** 2025-04-10

**Authors:** Ferdinand Nsengimana, Raoul Kamadjeu, Manassé Nimpagaritse, Eric Kezakarayagwa, Jonas Ngendakumana, Rémy Nimubona, Alain Yombouno, Godefroid Kamwenubusa, Rose Nkiko, Nestor Nkengurutse, Ndriakita Solonionjanirina, Hamady Ba, Beifith Kouak Tiyab, Liliane Nkengurutse, Edna Moturi, Dionis Nizigiyimana, Mame Selbee Diouf, France Begin, Joseph Nyandwi

**Affiliations:** 1 National Institute of Public Health, Bujumbura, Burundi,; 2 Health Emergency Preparedness and Response Team, UNICEF, New York, USA,; 3 City University of New York, School of Public Health and Health Policy, Department of Epidemiology and Biostatistics, New York City, USA,; 4 Africa Centres for Disease Control, Bujumbura, Burundi,; 5 Public Health Emergency Operational Centre, Ministry of Public Health and the Fight against HIV/AIDS, Bujumbura, Burundi, Kenya; 6 UNICEF Burundi, Bujumbura, Burundi, Kenya; 7 UNICEF Eastern and Southern Africa Regional Office, Nairobi, Kenya

**Keywords:** Mpox, Burundi, transmission dynamic, household transmission, secondary attack rate, breast milk, pregnancy outcomes

## Abstract

To address key knowledge gaps regarding mpox transmission and its effects on vulnerable populations, UNICEF commissioned an operational research study within the framework of the mpox outbreak response in Burundi. Through enhanced surveillance, this study, to be implemented by the Burundi National Institute of Public Health, will estimate mpox´s household transmission characteristics, including secondary attack rates and essential transmission parameters. The study will also evaluate the burden of mpox among children, its impact on pregnancy and pregnancy outcomes, and its association with breastfeeding, with the aim to generate data to optimize the mpox public health emergency response in Burundi and other affected regions.

## Introduction

Mpox, known previously as monkeypox [1], is a viral disease caused by the monkeypox virus (MPXV), an orthopoxvirus closely related to the human smallpox virus [2]. The natural reservoir of MPXV remains uncertain, but it is thought that small mammals such as rodents and monkeys in areas of Africa where the disease is endemic may harbor the virus [2]. There are two distinct genetic variants of MPXV known as clades and were previously named after their endemic presence in specific geographic regions [3]. Clade I, formerly called the Central African (Congo Basin) variant, is endemic to Central Africa, and Clade II, previously known as the West African variant, was predominantly endemic to Western Africa. Clade I infections are associated with greater disease severity and more pronounced rashes and have demonstrated, prior to the global emergence of clade IIb, increased human-to-human transmission compared to clade II [4].

While mpox transmission had been limited primarily to its endemic foci of Africa, cases related to laboratory animal travels and importation have been reported in non-endemic areas [5]. Things changed in 2022 with the rapid spread of MPXV to new geographic areas, resulting, by the end of 2022, to close to 85,000 confirmed infections in 109 countries [6]. This rapid global expansion of mpox prompted the declaration of a Public Health Emergency of International Concern (PHEIC) by the World Health Organization Director General [7]. During the 2022 mpox global epidemic, more than 90% of infections were linked to secondary transmission, the average age at the time of infection was above 30 years and infection occurred mainly in males who identified as males who have sex with males (MSM) [8,9]; this contrasted with the transmission pattern in endemic countries where the disease appeared to affect younger populations due to increased contacts with zoonotic sources and was associated with a higher case fatality in children. The increase in the global incidence of mpox due primarily to clade II, happened concomitantly with the spread of the disease in endemic provinces of the Democratic Republic of Congo (DRC), a country endemic to the disease. A subvariant of Clade I, known as Clade Ib, emerged in late 2023 and appeared to show increased human-to-human transmissibility [10]. Initially limited to DRC, the clade Ib mpox outbreak rapidly spread to several countries. As of 25 August 2024, six countries, including five in Africa (Rwanda, Burundi, Kenya, Uganda and Gabon), had reported cases directly linked to the outbreak in the DRC and the Clade Ib variant [6].

On August 13, 2024, the Africa Centres for Disease Control and Prevention (Africa CDC) declared the mpox outbreak a Public Health Emergency of Continental Security (PHECS) [11], the first such declaration since the agency’s inception in 2017. On 14 August 2024, the Director-General of the WHO declared mpox a Public Health Emergency of International Concern (PHEIC), the second such declaration for the disease in two years [12]. These declarations underscore the importance of mpox as a public health risk. In Burundi, a country on the eastern border of the Democratic Republic of the Congo (DRC), the first confirmed case of mpox linked to the outbreak in the DRC was reported on July 25, 2024 [13]. As of August 18, 2024, the country has reported 153 confirmed cases of mpox clade 1b, 30% of which were children under 15 years of age. As of 31 December 2024, close to 3000 cases of mpox have been confirmed in 46 of the 49 districts of the country: 51.8% were males, 20.6% below the age of five years; and those under the age of 15 accounted for 34% of all confirmed cases (Burundi SitRep - unpublished). Surveillance data in DRC demonstrated the high burden of mpox on children, with an estimated 80% of deaths occurring among children in the DRC. Factors such as malnutrition, the high prevalence of other infectious diseases, and limited access to healthcare services further increase the risk of severe outcome and mortality among children [14,15].

For decades, mpox remained a sporadic and relatively low-occurrence disease in endemic regions. However, the global outbreak driven by clade II, along with the emergence and rapid spread of clades Ia and Ib in both endemic and non-endemic areas of Africa, and the high mortality associated with mpox among children in the DRC, marked a significant shift. This unprecedented spread has exposed critical knowledge gaps regarding the disease´s clinical characteristics, transmission dynamics, risk factors, prevention, and treatments/medical countermeasures. Recognizing these gaps, calls for research to deepen the collective understanding of mpox to guide outbreak response efforts became louder. The WHO R&D Coordinated Research Road Map [16] identified ten immediate research next steps to contribute to control the mpox outbreak, which include obtaining additional data on mpox transmission and epidemiology, clinical outcomes and risk factors for severe disease; while African researchers, under the auspices of Africa CDC proposed an Mpox Research Group to advance mpox research agenda [17,18]. In this context, as part of its multisectoral approach to public health emergency preparedness and response, UNICEF commissioned a study on mpox to advance collective knowledge, with a particular focus on its impact on children, pregnant women, and underserved communities. The protocol was first drafted during a 5-day workshop conducted in Gitega (Burundi), from 8-11 October 2024, supported by UNICEF Burundi.

## Objectives

**Main objective:** the study aims to enhance understanding of mpox, with a particular focus on its impact on children, pregnant and lactating women.

**Specific objectives:** specifically, the research seeks to: 1) characterize household transmission dynamics and the role of children; 2) investigate the presence of MPXV in the breast milk of lactating women with confirmed mpox; and 3) assess pregnancy outcomes in women affected by mpox, as well as the health status of newborns born to mothers with confirmed mpox infection. This protocol is structured around the three specific objectives of the operational research.

## Study 1: mpox households transmission dynamics (enhanced mpox surveillance)

This protocol was guided by the World Health Organization (WHO), Investigation of Mpox Transmission - Study Protocol (unpublished, available upon request from mpox@who.int) [19].

**Objectives:** this aspect of the study aimed to explore the dynamics of mpox transmission in households, with the specific objectives below: 1) to estimate the transmission probability (secondary attack rate) of mpox in households, including children aged less than 15 years; 2) estimate mpox transmission parameters in households: incubation period, serial interval, reproduction number; 3) to determine the prevalence of mpox asymptomatic infection among members of a household with a confirmed mpox case.

**Figure 1:**
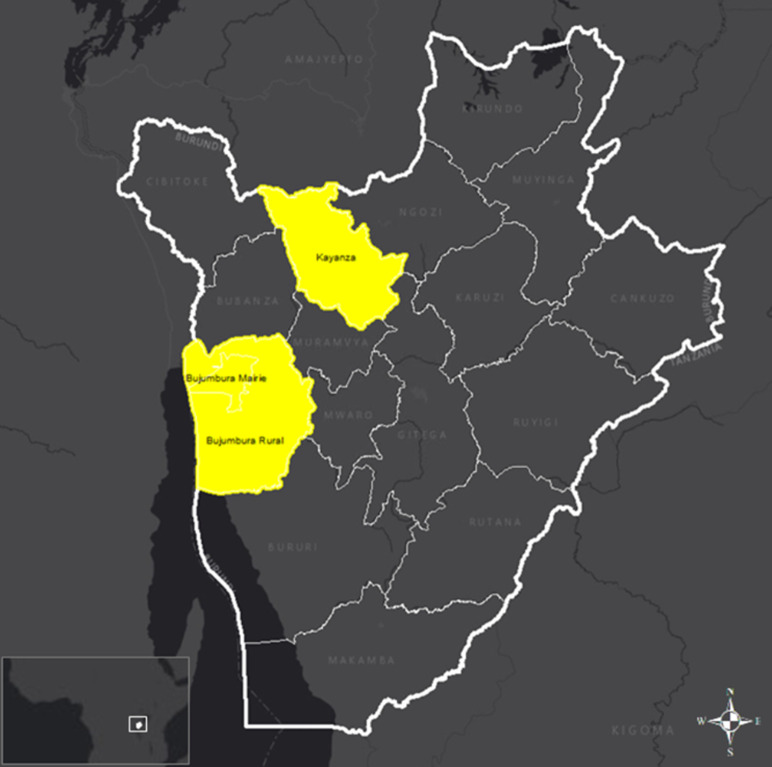
Burundi mpox study locations

**Study location:** the study will be conducted in the health districts of Bujumbura (urban) and Kayanza (rural). As of October 10, 2024, according to official mpox situation reports, these districts accounted for 60% and 3% of confirmed mpox cases, respectively. These sites were conveniently selected because they are locations for UNICEF-supported mpox response activities, have adequate human resources capacities for case assessment and follow-up, and have established infrastructure for sample collection, transportation, storage, and testing (Figure 1).

**Study design:** the study will employ an enhanced surveillance approach, investigating cases and their household contacts in line with the country´s National Directives for the Management of mpox [20] and the WHO´s Surveillance, Case Investigation, and Contact Tracing for mpox: interim guidance (November 27, 2024) [21]. Samples will also be collected from asymptomatic contacts to assess the extent of asymptomatic mpox transmission within households; this aspect is expanded in the relevant section below.

**Duration of the study:** the total duration of the study is estimated to be nine weeks, including six weeks for enrollment of household contacts, and three weeks of maximum follow-up time for all enrolled household contacts.

### Definitions of terms

**Confirmed case of mpox (primary case):** National Directives for the Management of mpox (DNPCM), a confirmed case of mpox is a person in whom mpox infection has been confirmed in the laboratory by the detection of unique viral DNA sequences using a real-time polymerase chain reaction (PCR) test and/or sequencing, as per mpox national surveillance standards.

**Household contact:** for the purpose of this study, a household contact is any person who resides in the same household as a confirmed case during the infectious period (defined below).

**Household:** for the purposes of this study, the following definitions of household are adopted: 1) ordinary household: a group of related or unrelated people, living together under the same roof, sharing meals together, and acknowledging the authority of a person called the head of the household. For the purposes of this study, selected households will have to include at least one child under the age of 15; 2) collective household: a group of people, generally not related, but living together in an institution for educational reasons. In the context of this study, this definition applies to primary and secondary boarding schools with children under 15 years of age.

**Infectious period:** corresponds to the period during which the confirmed case of mpox had a chance of infecting a household contact. This period is defined as the three weeks following the onset of symptoms, including mucocutaneous lesions or complete healing of the skin rash, in a person with confirmed mpox.

**Secondary case:** a household contact who develops clinical signs and symptoms of mpox during the follow-up period and is subsequently confirmed positive for mpox, with the primary case identified as the presumed source of infection within the household. The onset of symptoms for the secondary case must occur on or after the date of symptom onset in the primary case.

**Asymptomatic mpox case:** an individual who was exposed to a confirmed mpox case within the same household, has no prior history of mpox vaccination, and did not develop any signs or symptoms of mpox during the follow-up period. These individuals are identified as having an asymptomatic infection if they demonstrate a change in mpox antibody levels, suggestive of infection, based on analysis of at least two blood samples collected at least 21 days apart.

### Inclusion and non-inclusion criteria

***Inclusion criteria:*** the criteria for inclusion of cases, households, and household contacts are as follows:

***For confirmed cases (primary cases):*** 1) being a confirmed case of mpox (as per the national surveillance guidelines); 2) reside in one of the “study location” and in a household with at least one child under 15 years of age; 3) consent to participate in this study or, for minors, obtain the consent of the legally responsible adult.

***For the household:*** 1) the household hosted a confirmed case of mpox during the infectious period; 2) the head of the household consents to the inclusion of the household in the study; 3) at least one child aged less than 15 is a member of the household and resides in the household during the infectious period of the primary case.

***For household contacts:*** 1) reside in a household that meets the inclusion criteria for the household; 2) the household members provide informed consent for inclusion in the study, or, for minors (< 18 years), consent is obtained from the legally responsible adult.

***For contacts in collective households (boarding schools):*** 1) reside in a household that meets the inclusion criteria for the mpox case household; 2) Permission from the school authorities will need to be obtained for the monitoring of children in collective households. However, due to the absence of parents, samples for laboratory examinations will not be collected for follow-up, but in case of suspicion of mpox, the recommendations of mpox surveillance will be followed.

**Criteria for non-inclusion:** cases, households that do not meet the inclusion criteria.

**Sample size:** the study will use a sample of 100 confirmed cases of mpox (100 households). The sample size was conveniently determined using a non-probabilistic approach, dictated by the need to urgently generate evidence to inform the outbreak response while operating within the constraints of limited financial resources. Considering an average size of 5 people per household in Burundi (Demographic and Health Survey - DHS 2016 - 2017 data) [22], this translates into a total of 500 household contacts to be enrolled and monitored. The sample will be adjusted by a 20% refusal and loss to follow-up rate. The enrollment of confirmed cases and households will be done so that 70% and 30% of cases come from Bujumbura and Kayanza, respectively. In the city of Bujumbura, enrollment will be proportional to the contribution of each neighborhood to the total number of mpox cases reported in the city of Bujumbura, based on mpox surveillance data at the start of the study.

### Identification and enrolment of cases, households, and contacts

**Identification and enrolment of cases:** confirmed cases of mpox (cases) meeting the case definition will be identified through the mpox surveillance system. The number of confirmed cases (and households) in each district of Bujumbura will be proportional to the proportion of confirmed cases per district. At the beginning of the study, the distribution of confirmed cases of mpox by district will be derived from surveillance data. This list will be used to estimate the number of cases to be enrolled for each neighborhood. Cases will be recruited sequentially, by neighborhood. Recruitment will continue until the determined sample size, by neighborhood, is reached. The household of each case that consented to participate in this study will be enrolled and followed.

**Case assessment:** information on the clinical presentation of mpox will be gathered through a review of medical records from hospitalized cases in designated mpox treatment centers or other approved healthcare facilities. Additionally, an initial medical examination, at the time of enrolment in the study, will be conducted by a qualified healthcare provider from the research team using a standardized case form. Data collected will include demographic details, clinical symptoms, medical history, and potential mpox exposure.

**Case follow-up:** the clinical progression of confirmed mpox cases enrolled in the study will be assessed through a follow-up visit. For non-hospitalized cases, the assessment will be conducted during the weekly household visit. Hospitalized cases will be monitored by the study focal person within the mpox treatment centers or healthcare facilities, supplemented by a review of medical records. The monitoring process will track changes in clinical presentation and document the case’s final outcomes on the 21st day post-enrolment, including recovery, continued hospitalization, death, or loss to follow-up. All information will be recorded using a dedicated case monitoring form.

**Figure 2:**
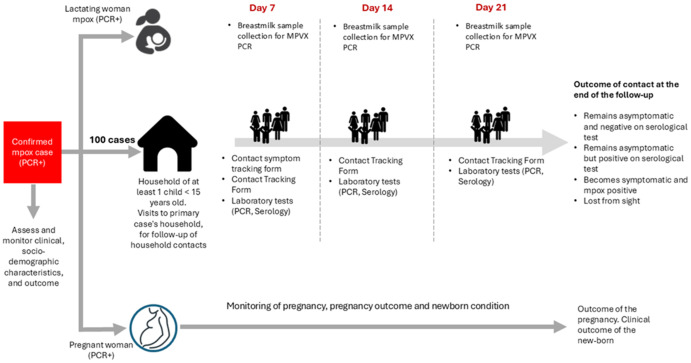
overview of study participants follow-up

**Households:** households of confirmed mpox cases meeting the inclusion criteria will be enrolled in the study upon obtaining consent from the head of the household. A home visit will be conducted as soon as possible after enrollment of the confirmed case. The primary objectives of the visit will be to document the household’s physical and socio-economic characteristics and to establish a comprehensive list of all eligible household members, including their names, ages (or dates of birth), and sex. During the initial household visit, data will be collected on key household attributes, including location, building structure, household composition, access to water and electricity, sanitation facilities, number of rooms, household size, and other socio-economic proxies. This information will be systematically recorded using a standardized household assessment form (Figure 2). Only households with at least one child under the age of 15 will be eligible for inclusion in the study. For this study, as defined earlier, household contacts will be limited exclusively to people living in the same household (regular or collective households) as the confirmed case, including during the infectious period.

**Figure 3:**
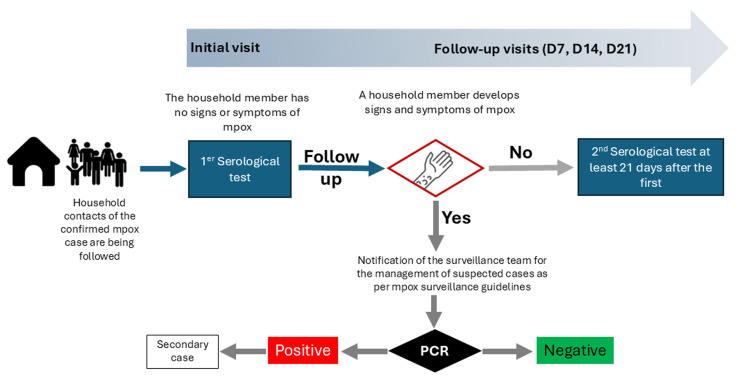
overview of household contacts monitoring and samples collection

**Identification and follow-up of household contacts:** household contacts will be identified from the list of contacts listed during the initial household visit. During the initial household visit, the investigation team will explain the purpose of the visit and conduct a physical examination of each household member. They will provide information on the risk of contracting mpox, outline its relevant signs and symptoms, and instruct household members to monitor their health and report any changes to the research team member without waiting for the scheduled weekly household visit. Additionally, the research team will explain the schedule of follow-up visits and provide clear guidance on how to contact them for any questions or to seek immediate medical care if symptoms suggestive of mpox develop. Efforts will be made by the data collection team to follow up with any household contacts who are absent during the initial visit (Figure 3).

**Follow-up visits in the household:** each household contact will undergo three follow-up visits on the 7th, 14th, and 21st days after the initial visit, to monitor their health status for mpox. During each visit, household contacts will undergo a clinical examination by a qualified healthcare provider member of the research team to assess the presence of any signs or symptoms compatible with mpox. If a household contact develops signs and symptoms suggestive of mpox during follow-up, they will be classified as “suspected mpox”, samples will be collected and tested for mpox as per the national mpox surveillance guidelines. Contacts who test positive for mpox (using PCR) will be classified as “secondary cases”. At that point, their participation in the study´s follow-up process will end, but they will continue to receive care and monitoring through the established mpox surveillance system.

### Laboratory procedures

**Sample collection for household contacts with suspected mpox:** household contacts who develop signs and symptoms suggestive of mpox (suspected cases) will undergo sample collection in accordance with the National Mpox Surveillance Directives [20] and the WHO Interim Guidance on Diagnostic Testing for MPXV [23]. The recommended specimen types include skin lesion samples, such as swabs of lesion surfaces, exudates, or lesion crusts.

**Sample collection for asymptomatic household contacts:** two blood samples will be collected from household contacts aged two years and older who remain asymptomatic throughout the follow-up period, with a minimum interval of 21 days between collections, for serological analysis to assess asymptomatic infection with mpox.

**Sample collection procedures:** health personnel performing the collection will wear adequate personal protective equipment (PPE) during sample collection, handling, and laboratory analysis procedures, in accordance with WHO Guidelines [23]. Hands will be cleaned before and after specimen collection, and the following PPE will be used by personnel collecting the sample: a disposable gown, gloves, eye protection (safety glasses, goggles, visor), and a surgical mask. A blood sample will be taken from all household eligible contacts using sterile equipment. Blood will be collected in labelled (name, date of collection, and contact identification number) tubes and transported in appropriate containers to the national reference laboratory.

**Sample processing, storage, and transport:** all samples will be collected according to standard procedures described in the National guideline and labelled with a coded identification number that will also be recorded on the interview questionnaire. The date and time of collection, the location, and the name of the person who took the sample will also be recorded in the interview questionnaire. All samples will be refrigerated (2 - 8°C) and transported to the national laboratory as quickly as possible. All samples transported will be triple-packed, labelled, and accompanied by the appropriate documentation providing information on the case. The date and time of collection, the conditions of transport, and the date and time of receipt by the study laboratory will be recorded for each biological sample collected. For blood samples: after treatment and separation of the serum, samples will be aliquoted before being frozen at -20°C or less. All personnel involved in the collection or transportation of samples will be trained in safe handling practices, appropriate personal protection, and spill decontamination procedures, as per the National Mpox Surveillance Directives and the WHO Interim Guidance on Diagnostic Testing for MPXV.

**More specifically, for serology, the following processes will be followed:** a minimum of four ml of blood will be collected in serum separator tubes (SST) through venipuncture. Labels, with personal identifiers, will be affixed to the SST tube; the data collection form will be completed with the correct participant information and matched to the SST tube; the data collection file will include: the contact personal identifiers, the collection date, the specimen ID (which will be the unique participant ID provided by the data collection team), and the submitter´s contact information; the blood collection tubes will be centrifuged at 1,000 - 2,000 Xg for 10 - 15 min, to separate serum from clotted blood. Aliquot serum or plasma in 0.5 mL aliquots in labeled 1.9 ml vials; the vials will be placed in cryovial storage boxes or larger volumes will be placed in shipping sleeves (e.g., Therapak Aqui-Pak Absorbent Sleeves (Fisher 22130043) [24] and seal in Ziploc® type plastic bag containing an absorbent pad/paper; the aliquots will be frozen at -20°C in the national lab for further analysis.

**Sample analysis:** all samples will be analyzed at the accredited national lab of the National Institute of Public Health.

**Polymerase chain reaction:** sample analysis will follow the procedures recommended by the WHO Diagnostic Testing for the Monkeypox Virus (MPVX) [23]. The RADI extraction kit will be used for nucleic acid extraction. The extracts will be amplified using the Altona kit (the FlexStar® monkeypox virus PCR Detection Mix 1.5) and then the RADI kit. RT-PCR will be the technique of choice with the CFX96 machine for nucleic acid amplification.

**Serology:** the aliquoted samples will be tested using the ELISA to detect orthopoxvirus IgG (IgG and IgM) using the technique described by Karem et al. [25] and Minhaj et al. [26]. Peak IgM is detected 2-3 weeks and IgG 3-5 weeks after exposure to an orthopoxvirus. Orthopoxvirus IgM is reliably detected 4-56 days and IgG >8 days after rash onset [25]. Considering the absence of vaccination in the country, and the relatively young age of participants (median age of cases in Burundi is 25 years) that exclude previous smallpox vaccination, the presence of IgM or IgG will be considered a marker of mpox infection.

### Data collection and management

**Data on the confirmed mpox case:** socio-demographic, clinical, and potential exposures and contacts during the infectious period will be collected from all cases enrolled in the study, using a Primary Case Form. (*See the WHO investigation of mpox transmission - study protocol*).

**Household data:** household data will be collected to help quantify the economic characteristics of the household, proximity to treatment facilities, and the number of occupants, using a household form.

**Household contacts data:** household contact data will be collected to detect any reported infections in the household case and to identify contacts who may become secondary cases following exposure to the case. Household contacts will constitute the base population for the calculation of secondary infection rate, secondary clinical attack rate, serial interval, basic reproduction number (R0), and incubation period.

### Data collection tools

**Case investigation form:** a detailed case investigation form will be used to gather information on all confirmed mpox cases. It will be completed by the healthcare professional (registered nurse or doctor) who will assess the individual, and will contain sociodemographic information (age, gender, occupation, etc.), medical history (pre-existing conditions, history of vaccination against smallpox and monkeypox), information on vaccine doses received during routine vaccination for eligible mpox cases (children under 5 years of age), and information on exposure in the 21 days prior to the onset of symptoms, either to an infected animal or human, or to contaminated material (type of contact, place where the contact took place, relationship with the contact person, etc.).

**Household identification and assessment form:** a household form will be used for household identification and assessment. This form will be completed by one of the interviewers and includes: the geographical location of the household (district, commune, district, block, address, geolocation coordinates...), the name of the head of household, the number of occupants, and details that can be used to assess the socio-economic status of the household (number of rooms, building materials, water supply, lighting source).

**Initial contact investigation form:** will be used for each person in the case’s household.

**Contact person tracking form:** for household members on D7, D14 and D21.

**Contact person symptom log:** self-completed by the contact person for 7 days for assessment of signs and symptoms consistent with mpox.

A summary of all data collection tools is provided in supplemental material (Annex 1, Annex 2). These forms will be translated into Kirundi (the national language of Burundi), pre-tested and transported to the web platform KoboToolbox [27]. The data will then be collected using the KoboCollect app installed in Android tablets. Data collected through KoboCollect are synchronized and automatically backup to a remote server for backup. A data quality monitoring system will be implemented to ensure the continuous integrity of the data collected. The data manager and study supervisors will oversee quality control throughout the data collection process. All data collectors will receive comprehensive training on study tools, data collection and interview techniques, and fundamentals of the study´s ethics to ensure adherence to ethical standards.

**Annex 1:** type of samples collected from house contacts and lactating women

Person	Type of sample	Purpose/goal of collection	Timing of collection
Household contact suspected of mpox	skin lesion samples, such as swabs of lesion surfaces, exudates, or lesion crusts	Laboratory confirmation of mpox	As soon as a skin rash appears
Household contacts aged 2 years and above who remain asymptomatic for mpox	Blood	To assess the serological status and dynamics of mpox antibodies.	Two samples will be collected: the first one at the time of enrollment or as soon as possible after enrolment; and at the 3rdvisit, at least 21 days after the first sample
Lactating women	Breast milk	To assess the expression of MPXV material in breastmilk	Weekly, at most three times, if breast milk is positive (PCR), at most twice, if two consecutive negative samples

**Annex 2:** summary of data collection tools

Form Title	Form Purpose	Person filling out the form	Timing of data collection
**Cases**			
Initial case investigation Form	For information related to the confirmed case of monkeypox.	To be completed by the healthcare worker of the research team or the provider at the mpox treatment center	Day of enrolment into the study
Case tracking form (integrated with the study's case investigation form)	For 28-day follow-up or at hospital discharge (or in the event of death) of confirmed cases of monkeypox, to record outcome of the case	To be completed by the study team members responsible for monitoring in the household	28 days after enrollment or after hospital discharge or death
**Household**			
Household form	Collect information related to the household (location, physical characteristics, socio-economic characteristics, number,and basic demographic characteristics of residents)	To be completed by the study team members responsible for monitoring in the household	At initial contact with the household, after obtaining consent from the head of the household.
**Household contacts**			
Contact initial investigation Form	For household contacts of confirmed monkeypox cases.	To be completed by a member of the research team	Day of enrolment of contact into the study
Contact tracking form	For active follow-up of household contacts	To be completed by a member of the research team	At 7-, 14-, and 21-daypost-enrolment
**Pregnant women confirmed with mpox**			
Initial case investigation Form Follow-up of pregnant women and newborns	Pregnancy follow-up according to the prenatal consultation schedule Findings from obstetric ultrasound (maximum 3, depending on the age of pregnancy at the time of diagnosis mpox Clinical evaluation of newborns	To be completed by a member of the research team	During pre-natal visits and at the outcome of the pregnancy.
**Lactating women confirmed with mpox and their children**			
Initial case investigation Form Lactating women form Case tracking Form (integrated with the study's Case investigation form)	To assess the clinical status of lactating women with mpox To assess the clinical status of their child To collect breast milk sample To monitor the clinical evolution of the breast lactatingwomen and their child	To be completed by a member of the research team	At 7-, 14, and 21-daypost-enrolment

**Data analysis plan:** the analysis will focus on the following aspects.

**Descriptive statistics:** a frequency analysis will be performed for the main sociodemographic data (age, sex, occupation, health status, etc.) of cases, contacts, clinical characteristics of cases (onset of symptoms, type of symptoms, co-infections, outcomes) and information on the exposure of cases and contacts (epidemiological link, type of contact, exposure setting, relationship with the contact person, etc.). etc.).

**Main analyses:** the main analysis of the study will focus on the following parameters.

**Incubation period:** the time between exposure and the onset of symptoms in a case. Cases for which information on the date of exposure and date of symptom onset is available will be included in the descriptive analysis; in some cases, a plausible range of exposure dates will need to be given. The distribution of observed incubation periods will be described. The Bayesian interval censorship model, with right truncation, will be used to estimate the distribution of observed incubation periods, to account for uncertainty about the precise timing of exposure.

**Serial interval:** the time between the onset of symptoms in a primary case and the onset of symptoms in secondary cases infected with the primary case. Cases for which the dates of symptom onset (either constitutional symptoms or rash) are precisely known for both primary and secondary cases and where the epidemiological link is clear will be included in the descriptive analysis. The distribution of the observed time interval for each data pair will be calculated and described. Bayesian methods, with right-hand truncation, will be used to estimate the distribution of the serial interval for the known primary and secondary case pairs.

**Household secondary attack rate (SAD):** the proportion of exposed individuals in the household of the confirmed case, who develop disease during the incubation period following contact with a primary case. Data will be collected by recording the number of new symptomatic infections among contacts of primary cases within 21 days of contact follow-up. The SAD will be calculated by dividing the number of secondary cases by the total number of susceptible contacts followed. The results will be accompanied by confidence intervals to indicate the precision of the estimates.

**Asymptomatic secondary attack rate (SAD) in households:** the proportion of exposed individuals in the household of the confirmed case, with no history of mpox vaccination, not showing signs or symptoms of mpox during the incubation period following contact with a primary case, but demonstrating a change in mpox antibody dynamics suggestive of asymptomatic infection, based on at least 2 blood samples collected at a minimum of 21 days apart, will be used to calculate the asymptomatic secondary attack rate. Data will be collected based on at least 2 blood samples collected at least 21 days apart from household contacts who do not develop clinical signs or symptoms of mpox during follow-up, hence, have no sample collected for PCR. The SAD in asymptomatic individuals will be calculated by dividing the number of asymptomatic secondary cases, with a change in antibody dynamics suggestive of asymptomatic mpox infection, by the total number of susceptible contacts followed, remaining asymptomatic to mpox.

**Reproduction number (R0):** represents the average number of secondary infections produced by an infected individual in a fully exposed population. The data required includes the number of secondary cases from a primary case and contact patterns. The appropriate mathematical model, taking into account the generation time and its appropriate distribution, will be used to estimate R0.

**Risk factors for mpox in contacts:** all contacts who are members of the same household as the confirmed mpox case will be included, divided into three groups, those who have developed mpox disease, those who have developed asymptomatic infection (defined as positive serial mpox serology without symptoms or skin lesions), and those who have developed neither. Data permitting, multivariate logistic regression analysis will be used to identify risk factors for progression to MPXV infection in contacts. Predictive factors that will be explored will include demographics, exposure information, and clinical characteristics.

## Study 2: mpox and lactation

### Introduction

The relationship between mpox and breastfeeding remains an area of ongoing research, and it is not yet known whether the MPXV or mpox antibodies are present in the breast milk of infected lactating mothers. The US CDC advises delaying breastfeeding until the risk of transmission to the infant is minimized, specifically, when all lesions have healed, scabs have fallen off, and a fresh layer of intact skin has formed [27]. In contrast, the WHO recommends assessing infant feeding practices on a case-by-case basis, taking into account the mother’s overall health and the severity of her illness, as these factors may influence the risk of mother-to-child transmission [28].

**Objectives:** this component of the study will evaluate, through PCR, the presence of mpox virus (MPXV) genetic material in the breast milk of breastfeeding women confirmed to have mpox and will assess the clinical presentation and outcomes of their breastfed children. However, the detection of viral genetic material does not necessarily indicate the presence of a viable, infectious virus. Confirming viral viability would require viral culture, which is essential to determine the potential risk of transmission through breast milk. Unfortunately, due to laboratory constraints, viral culture could not be performed as part of this study.

### Methodology

**Study type:** this prospective study will monitor the health status of breastfeeding women confirmed to have mpox and collect breast milk samples at seven-day intervals for laboratory analysis (see sample collection section). Additionally, the clinical status of breastfed children will be closely monitored.

**Place:** see study location in study 1.

**Target population:** lactating women confirmed positive for mpox, and their breastfed child(ren).

**Sample size:** the number of breastfeeding women infected with mpox will be estimated based on the total number of confirmed cases among breastfeeding women since the onset of the epidemic, which is currently estimated at 15. Due to the anticipated limited number of lactating women confirmed with mpox, all breastfeeding women confirmed to have mpox and identified during the study period will be enrolled.

### Inclusion and non-inclusion criteria

**Inclusion criteria:** breastfeeding women confirmed positive for mpox according to National Directives, with no visible lesions on the nipples, and who provide informed consent for participation. Their breastfed child will be enrolled as a contact and monitored for clinical presentation and outcomes.

**Non-inclusion criteria:** lactating women with mpox lesions on the nipple, or lactating women refusing to participate in the study or providing breastmilk samples.

**Figure 4:**
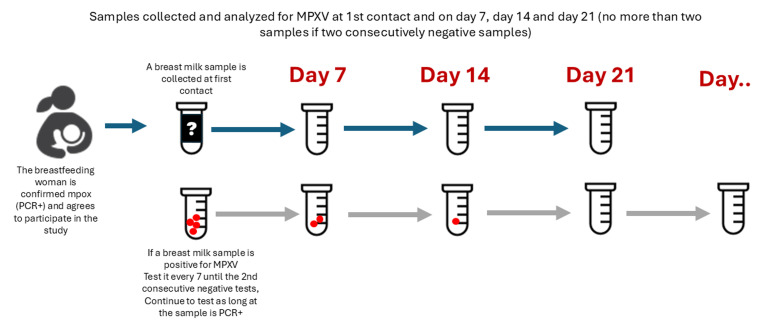
lactating women monitoring and sample collection

**Identification, enrollment, and follow-up of participants:** lactating women who are confirmed with mpox will be identified through the mpox surveillance system, mpox treatment centers, or health facilities. A lactating woman may be enrolled in the study both as a primary case and as a breastfeeding mother. In this scenario, she will be monitored in both capacities, and her household will be included for follow-up. However, if a lactating woman with confirmed mpox is not enrolled as a primary case, she will be monitored individually without enrolling the household. All children breastfed by the infected woman will be considered has her contacts and followed according to the contact follow-up procedures (see Household contacts follow-up section) (Figure 4).

**Data collection:** the case follow-up form will be used for the follow-up of breastfeeding women. An annex to the form will collect information on the health status of the child.

### Laboratory procedures for lactating

**Sample collection:** breastmilk from lactating women will be collected following the best practices for breastmilk collection and storage described by McGuire et al. [29]. Breast milk samples will only be collected from people without visible lesions on the breasts, as the lesions may result in the presence of viral virus/genetic material in the collected breast milk.

**Breast cleaning:** since the primary research relates to whether genetic material of the mpox virus is present in the milk, the breast will be thoroughly clean before milk collection, particularly since the pathogen may be transmitted into milk through touches, cloths or respiratory droplets. After donning face covering and a glove on the hand that will clean the breast, the research personnel (a woman) in charge of breastmilk collection will clean the “study breast” thoroughly with aseptic wipes. The purpose of this step is to physically remove all potential contaminants.

**Milk collection:** with a newly gloved hand, the research personnel will gently press the breast to express 1 ml of milk into the sterile collection container. The goal is to obtain a “clean catch” sample that drips or squirts directly from the nipple into the sterile container.

**Milk storage:** the collected milk will be placed immediately in the cold box and transported to the laboratory on ice (2 - 8°C) where it will be frozen at âˆ’20°C. Instructions will be given not to freeze/thaw the breast milk sample.

**Lab processing of milk samples for PCR:** breastmilk samples will be processed following a technique described in the supplemental material (Annex 3).

**Annex 3:** analysis of breast milk samples in the laboratory

This appendix describes the procedures for the preparation and PCR testing of breast milk samples. All analyses were carried out at the laboratory of the National Institute of Public Health (INSP). **Sample preparation** Milk should be pre-processed to reduce its lipid and cellular component content. Centrifuge the milk at 2500g (3500rpm) for 15 min Recovering the supernatant Dilute the sample (supernatant) to 1/10 **Extraction** 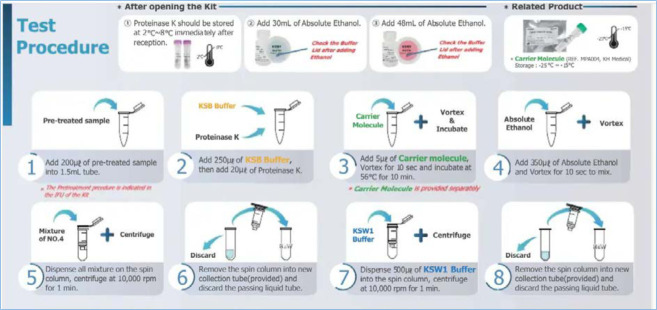

**Data analysis plan:** the following analyses will be carried out: proportion of breast milk samples positive for mpox using PCR; clearance time of MPXV from breast milk; rate of clinical mpox infections in breastfeed children; association between breastmilk PCR test and mpox infection in the breastfed child.

**Ethical considerations:** refer to the general ethical considerations section.

## Study 3: pregnancy, newborns and mpox

### Introduction

Data on the impact of mpox on pregnancy are scares, particularly in areas with endemic transmission and within the context of the current mpox PHEs affecting countries in Africa, characterized by active human-to-human transmission [30]. Data from smallpox reveal high rates of miscarriages and other adverse pregnancy outcomes.

Objectives: to assess the outcome of pregnancies in pregnant women affected by mpox and the health status of the newborn of pregnant women confirmed positive for mpox.

### Methodology

**Study type:** a combination of prospective and retrospective study design.

**Study location:** see the study location in study 1.

**Target population:** women who have contracted mpox during pregnancy, since the beginning of the outbreak, regardless of the age of pregnancy.

**Sample size:** given the small number of pregnant women, the study will enroll all pregnant women confirmed positive for mpox since the beginning of the epidemic, irrespective of the age of pregnancy at the time of mpox diagnosis.

### Inclusion and non-inclusion criteria

**Inclusion criteria:** pregnant women diagnosed positive for MPXV during pregnancy, since the beginning of the outbreak, residing in Bujumbura or Kayanza districts, and agreeing to participate in the study.

**Non-inclusion criteria:** individuals who do not meet the inclusion criteria.

**Identification and enrolment of participants:** pregnant women confirmed with mpox will be identified through the mpox surveillance system, mpox treatment centers, or health facilities.

**Figure 5:**
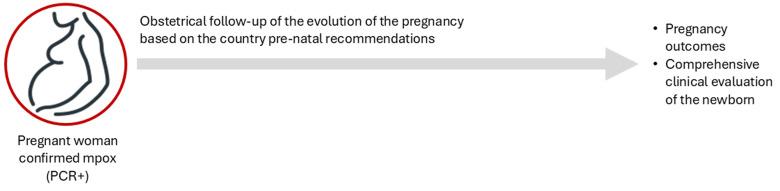
pregnant women monitoring

**Data collection:** pregnant women who have consented to this study will be followed up until the end of the pregnancy. Follow-up will be done according to the prenatal consultation schedule. At delivery, a thorough examination of the newborn’s state of health will be carried out by a dedicated pediatrician. Pregnant women admitted to an mpox treatment center in the city of Bujumbura for mpox before the start of the study and since the beginning of the epidemic will be traced and the maternal and neonatal outcome recorded (Figure 5).

**Data analysis plan:** the following analysis will be carried out: distribution of the clinical presentations of mpox in pregnant women at enrolment (severity of cases); distribution of pregnancy outcomes in pregnant women infected with MPXV (normal delivery, preterm birth, neonatal mortality, spontaneous abortion); description of the clinical presentation of newborns of mothers infected with MPXV.

**Ethical considerations:** refer to the general ethical considerations section.

## Data processing and archiving

Article 12 of the legal framework for statistics in Burundi stipulates that: (i) individual data from statistical surveys and censuses are protected in the same way as the individual freedoms of citizens. In any case, they may not be used for the purposes of prosecution or fiscal or criminal punishment except in cases expressly provided for by law. Under no circumstances may the individual data collected be used for any purpose other than to disseminate or publish aggregated statistical results. The data will be collected on tablets using the KoboCollect App [27]. Before the analysis, the database will be checked and its consistency checked. To ensure data quality, the data entry form will include internal quality checks to identify data that appears inconsistent, incomplete, or inaccurate. Study participants will be identified by a unique participant number in the database. The principal investigator is responsible for ensuring that data collection and capture are accurate and complete and for keeping all screening forms, case registration form, and completed Subject Identification Code List in a secure location. Daily data consistency and quality checks will be conducted and issues identified discussed and addressed. Access will be granted to individuals authorized by the Principal Investigator only.

## Ethical considerations

All study participants will provide informed consent through a signed consent form written in the local language. For participants who cannot read, the consent form will be read aloud by the interviewer, and consent will be obtained through either a signature or a fingerprint mark. Participation in the study is entirely voluntary, and participants will be informed of their right to withdraw at any time without consequence. For minors, consent will be obtained from a parent or legal guardian, in accordance with ethical guidelines for research involving children. In cases involving collective households, such as boarding dormitories, administrative permission will be secured from the relevant institutional authorities before enrollment. Prior to obtaining consent, the research team will comprehensively explain the study’s objectives, procedures, potential risks, and anticipated benefits to all prospective participants or their legal guardians. Specific consent will be sought for all sample collections, including blood draws and other biological specimens. To minimize unnecessary distress, no venipuncture will be performed on children under the age of two. To ensure the privacy and confidentiality of study participants, no names or personally identifiable information will be included in the database or datasets used for analysis. All collected data will be securely stored, with access restricted to authorized study personnel. Data collected using KoboCollect [27] will be encrypted and stored on a password-protected server, in compliance with international best practices for data protection. Daily data consistency and quality checks will be conducted, and any identified issues will be promptly addressed to maintain data integrity. The study will seek and obtain ethical approval from the Burundi National Ethical Committee and adhere to all administrative and regulatory requirements set by the government of Burundi for conducting research. Additionally, all study procedures will align with international ethical standards, including the Declaration of Helsinki [31] and the principles of Good Clinical Practice (GCP) [32]. Participants will receive appropriate medical care and referral services as needed, ensuring that study participation does not compromise their health or well-being. Where appropriate and feasible, mechanisms will be considered to inform participants or communities about the overall findings of the study, while maintaining individual confidentiality. The study received ethical approval from the National Ethics Committee for the Protection of Human Subjects of Biomedical and Behavioral Research, under decision CNE/34/2024 (25 Nov 2024).

## Dissemination of the study results

The findings of this study will be shared through multiple channels to maximize visibility, impact, and engagement with key stakeholders; results will be presented at relevant national and international conferences, as well as public health forums, to engage experts, policymakers, and practitioners in the field; to contribute to the scientific literature and provide evidence-based insights, study findings will be submitted for publication in high-impact peer-reviewed journals; key findings may also be disseminated through media platforms, ensuring broader public awareness and understanding of the study’s implications; all dissemination activities will strictly adhere to ethical guidelines, ensuring data confidentiality, participant privacy, and compliance with applicable research regulations.
